# Peripheral Neuropathy as a Risk Factor for Developing Cardiovascular Disease in Diabetic Patients

**DOI:** 10.7759/cureus.11922

**Published:** 2020-12-05

**Authors:** Ghassan AlGhamdi, Hisham Saati, Enad Almotairi, Basil S Alsofiani, Abdulhalim J Kinsara

**Affiliations:** 1 Medicine, King Saud Bin Abdulaziz University for Health Sciences College of Medicine, Jeddah, SAU; 2 Internal Medicine, King Saud Bin Abdulaziz University for Health Sciences College of Medicine, Jeddah, SAU; 3 Cardiology, Ministry of National Guard - Health Affairs, King Saud Bin Abdulaziz University for Health Sciences, College of Medicine, Western Region (COM-WR) King Abdullah International Medical Research Center, Jeddah, SAU

**Keywords:** diabetes mellitus, diabetic peripheral neuropathy, cardiovascular disease, neuropathic disability score, saudi arabia

## Abstract

Background

Type 2 diabetes mellitus (DM) is prevalent in developing countries and is associated with many comorbidities, including diabetic peripheral neuropathy (DPN) and cardiovascular disease (CVD). In this study, we assessed and evaluated the association between DPN and CVD.

Methods

The study is a cross-sectional study that included DM patients who are attending DM primary care clinics. We evaluated each patient and collected epidemiological data, the physical examination findings, including cardiovascular status, and the presence of DPN. DPN was assessed with the neuropathic disability score (NDS), and it was considered present if the score was 5 or higher. The age and the levels of blood glucose, HbA1C, and plasma total cholesterol were recorded.

Results

The study included 116 DM, the mean age was 64.5±15 years (19 to 84 years) and the majority (61.2%) of the sample were male. The sample was divided into two groups: patients with DM only and patients with DM and CVD. The CVD group had a higher NDS score compared to the non-CVD group (P=0.006). The result indicated that for the CVD group, the prevalence of DPN was higher (50.8%) as compared to the non-CVD group (30.2%) (P=0.041). DPN was also associated with the acute coronary syndrome (ACS) (P = 0.013) but not heart failure (HF) (P=0.427). In addition, the HbA1C was significantly higher in patients with DPN (P=0.0345).

Conclusion

Our findings indicate that DPN was more prevalent in the CVD group and, in particular, in the group with ACS. The evidence provides support for the association between DPN and CVD.

## Introduction

Type 2 diabetes mellitus (DM) is prevalent globally, and in Saudi Arabia, the prevalence rate is one of the highest [1]. According to the World Health Organization, the prevalence of diabetes in the total population of Saudi Arabia is 14.4% [2]. Diabetic peripheral neuropathy (DPN) is a frequent complication associated with DM. DPN is defined as “the presence of symptoms and/or signs of peripheral nerve dysfunction in people with DM after the exclusion of other causes [3]. Another complication of DM is cardiovascular disease (CVD). A study reported that DM increases the chance of developing CVD two-fold, specifically acute coronary syndrome (ACS) or heart failure (HF) [4].

We propose that the shared pathophysiological mechanisms will result in a close association between the two complications. There are only a few studies available regarding the association between DPN and CVD. A study in 2014, conducted in the United Kingdom with 13,043 individuals, reported an association between patients with DPN and an increased risk of CVD [5]. No local studies are available regarding this subject.

The research will provide additional evidence of an association between CVD in DM patients with DPN.

## Materials and methods

The study was a cross-sectional study with a sample of 116 DM patients, all over 18 years old and irrespective of gender. Patients with one of the following risk factors were excluded from the study: connective tissue disorders, alcoholism, vitamin B12/folate deficiencies, and primary neurologic disorders (previous spinal injury, a history of lumbar or cervical disc disease, carpal tunnel syndrome, and inherited neuropathy).

The study was approved by the local institutional review board. A standardized data collection form was completed after obtaining written informed consent from the participant. The form collected data about the participant’s age, gender, blood pressure, blood glucose, glycated hemoglobin (HbA1C), plasma total cholesterol, low-density lipoprotein, and the presence of CVD with its type.

DPN was recorded as a neuropathy disability score (NDS). An NDS score of 5 or more was indicative of DPN. Sensory, motor, vibration, and temperature neuropathies were assessed. The data was stored on Microsoft Excel (Microsoft Corporation, Redmond, WA) and analyzed with the Statistical Package for the Social Sciences (SPSS) version 22 (IBM Corp., Armonk, NY). DPN was the independent variable, and the outcome variable was the proportion with CVD. The qualitative variables are presented as frequency and percentage, and the quantitative variables as mean and standard deviation, if normally distributed, or if skewed, the median and 25th to 75th percentile. A chi-square test was used for the qualitative variables and a Mann-Whitney U for quantitative variables. A P-value of < 0.05 was considered significant, with a confidence interval (CI) of 95%.

## Results

In total, 116 participants who met the inclusion criteria were enrolled in this study, with the majority (61.2%) male. The mean age was 64.5±15 years (19 to 84 years). The sample was assigned to two groups: a group with DM and CVD (49.2%) and the non-CVD group.

The overall DPN prevalence rate was 41.4%. The CVD group had a significantly higher NDS score (6±5) as compared to the non-CVD group (6±1) (P=0.006). The results indicated that the prevalence of DPN in the CVD group was (50.8%) as compared to the non-CVD group (32.2%) (P=0.041) (Table 1).

**Table 1:** Comparison between DM with/without CVD DM: diabetes mellitus; CVD: cardiovascular disease

	DM with CVD (n=57)		DM without CVD (n=59)		P-value
Age (years)	65±17		64±15		0.21
Gender: Male	37	64.9%	34		4.51
Acute coronary syndrome	38	63.2%	0		> 0.001
Heart failure	26	45.6%	0		> 0.001
Neuropathy disability score (NDS)	6±5		6±1		0.006
Diabetic peripheral neuropathy	29	50.8 %	19		0.041
Blood glucose	8.7±7.6		8.8±3.5		0.987
Glycated hemoglobin (HbA1C) (mmol/mol)	7.7±1.7		8.3±1.5		0.006
Low-density lipoprotein (LDL) (mg/dL)	2±0.8		2.3±1.2		0.37
Total cholesterol	3.5±1.5		4±1.5		0.01

We also found a significant association between the presence of ACS and the development of DPN (P=0.013), representing 58.33% of the group with DPN. However, HF had no significant association with DPN (P=0.427). A higher HbA1c score was associated with a higher prevalence of DPN (P=0.0345), (Tables 2-3). 

**Table 2:** Characteristics of DM patients with DPN DM: diabetes mellitus; DPN: diabetic peripheral neuropathy

Variables	DPN				P-value
	Yes (n=48)		No (n=68)		
Gender: Male	32	45.07%			0.311
Age (years)	64.9± 9.0		59.6 ± 14.9		0.0610
Acute coronary syndrome	21	58.33%	15		0.013
Heart failure	9	34.62%	17		0.427
Blood glucose	11.2± 4.9		9.15 ± 3.9		0.0127
Glycated hemoglobin (HbA1c) (mmol/mol)	8.4 ± 1.8		7.7 ± 1.4		0.0345
Low-density lipoprotein (LDL)	2.3 ± 0.8		2.3± 0.9		0.9263
Total cholesterol	3.9 ± 0.96		3.9± 1.2		0.7922

**Table 3:** Comparison between patients with DM and DPN with or without CVD DM: diabetes mellitus; CVD: cardiovascular disease; DPN: diabetic peripheral neuropathy

Variable	DPN +DM +CVD (n=29)	DPN +DM (n=19)	P-value
Gender: Male	18	14	0.404
Age	65.2 ± 8.4	64.4 ± 10.3	0.7354
Acute coronary syndrome	21	0	0.000
Heart failure	9	0	0.007
Blood glucose	11.9 ± 5.6	10.2 ± 3.5	0.5133
Glycated hemoglobin (HbA1C)	8.0 ± 1.8	8.9 ± 1.7	0.0876
Low-density lipoprotein (LDL)	2.2 ± 0.74	2.5 ± 0.86	0.4542
Total cholesterol	3.8 ±.86	4.1 ± 1.0	0.3536

## Discussion

In the current study, the rate of DPN was significantly higher in the CVD group and specifically in participants diagnosed with ACS. Many of the patients were unaware that they had DPN. We also found that DPN was associated with a higher HbA1c score and a higher level of blood glucose. However, the other risk factors (gender, age, LDL, cholesterol), although numerically higher in the group with DPN, were not statistically significant.

Three studies are available reporting an association between DPN and increased CVD events [5-7]. Although they used different methods to assess DPN, they demonstrated an association between the two DM complications. One of the studies used the same neuropathic disability score (NDS) to assess DPN and reported an association [6]. Another cohort study, with 13,043 patients, reported that DPN was associated with an increased risk of a first CVD event in patients diagnosed with DM [5]. A prospective cohort study investigating DPN as a predictor for developing CVD in DM patients reported that patients with DM and CVD at baseline had an increased risk of developing DPN (Table 4) [7].

**Table 4:** Summary of similar studies DM: diabetes mellitus; CVD: cardiovascular disease; DPN: diabetic peripheral neuropathy; MI: myocardial infarction

First Author	Publication Date	Sample Size	Study Design	Conclusion
Dimitrios Baltzis [6]	Aug 23, 2016	82	Cross-sectional	DPN associated with MI
Jack RW Brownrigg [5]	Aug 5, 2014	13,043	Cohort	DPN associated with increased CVD risk
Juan Ybarra-Muñoz [7]	Dec 19, 2014	267	Cohort	CVD associated with increased DPN risk

A limitation of this study was the small sample size. Larger sample size would have enabled us to assess other risk factors for CVD in addition to DPN.

## Conclusions

The rate of DPN in the CVD group was noticeably higher as compared to the non-CVD group. ACS was significantly associated with DPN but HF was not associated. DPN had uncontrolled risk factors and careful clinical assessment might indicate a higher incidence than what is currently known.
